# Short Jute Fiber Preform Reinforced Polypropylene Thermoplastic Composite: Experimental Investigation and Its Theoretical Stiffness Prediction

**DOI:** 10.1021/acsomega.3c01533

**Published:** 2023-06-28

**Authors:** Nazrima Sultana, Mahmudul Hasan, Ahasan Habib, Abu Saifullah, Abu Yousuf Mohammad Anwarul Azim, Shah Alimuzzaman, Forkan Sarker

**Affiliations:** † Department of Fabric Engineering, Bangladesh University of Textiles, Tejgaon, Dhaka 1208, Bangladesh; ‡ Central Quality Control Department, Janata Jute Mills Limited, Akij Holdings Limited, Dhaka 1212, Bangladesh; § Department of Textile Engineering, Dhaka University of Engineering & Technology, Gazipur 1700, Bangladesh; ∥ Advanced Polymers and Composites (APC) Research Group, School of Mechanical and Design Engineering, University of Portsmouth, Portsmouth PO1 3DJ, U.K.

## Abstract

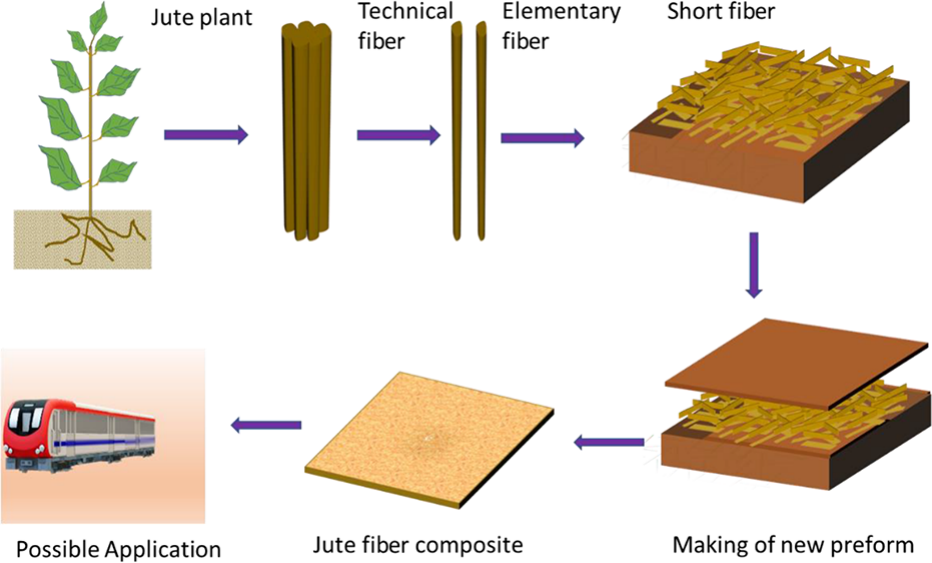

Natural-based lignocellulose fibrous materials can be used as a sustainable alternative to conventional fossil-based fibers such as glass fibers, in lightweight fiber-reinforced thermoplastic composites for marine, automotive, aerospace, or other advanced applications. However, one of the main challenges in using natural fiber-based thermoplastic composites is the low mechanical performance of composite structures. This can be improved significantly through the development of an optimized novel fiber architecture with enhanced fiber packing properties, following a low-cost production process. In this context, this study demonstrates a less energy-consuming and cheaper manufacturing process, for developing highly individualized short jute fiber-based dry fiber preform architecture, with an improved fiber packing property. Short jute fibers were chemically treated with alkali and PVA sizing treatments in the processing of new fiber preform architectures, and they were used in manufacturing of ultimate short jute fiber/polypropylene (PP) thermoplastic composites. The newly developed short fiber thermoplastic composites showed a significant improvement in mechanical properties (tensile, flexural, and impact) compared to any other natural fiber architecture-based (woven, knitted, nonwoven, unidirectional, etc.) composites found in the literature. Due to the use of new fiber architecture, the developed composites’ fiber content was observed to increase. In addition, the compatibility of jute fibers with the polypropylene matrix was strengthened with the application of chemical treatments on highly individualized jute fibers. These reasons were responsible for the enhancement of mechanical properties of developed composites. Micromechanics of the fibers in composites were evaluated using the modified rule of the mixture and Halpin–Tsai equations for stiffness prediction of the composites in order to develop a theoretical understanding of newly developed composites’ mechanics. It is thought that the improved mechanical performance of short jute fiber/PP thermoplastic composites can extend the use of these composites in many load-demanding applications, wherein normally synthetic fiber composites are used.

## 1 Introduction

Recently, an increasing focus is found in research and engineering fields on the development of fiber-reinforced polymeric composites as viable structural materials for various industrial applications, such as aerospace, marine, leisure, automotive, construction, and sports,^1,2^ because of their lightweight, excellent specific strength and stiffness, durability, creep, heat, fatigue, and corrosion resistances. It is known that fiber-reinforced polymer composites consist of at least two constituents: (1) the reinforcing fiber element and (2) the polymer matrix. The mostly used reinforcing fibers include glass, carbon, aramid, Kevlar, etc., which are being originated from fossil-based nonbiodegradable synthetic sources.^3^ Considering the current drive for environmental management and sustainability, government’s strict legislations at different parts of the world to get rid of CO_2_ emission significantly within 2030 and very recent COP27 guidelines, it is urgently needed to replace the use of synthetic nonbiodegradable fibers with nature-based biodegradable fibers for composite applications.

In this regard, natural fibers such as, flax, hemp, jute, sisal, kenaf, etc., are getting huge attention from manufacturers and researchers since they are from biodegradable origins and exhibit comparable mechanical properties, lower cost, densities, and hence, production of lightweight structures.^4^ In automotive industries, environment friendly sustainable composites are being manufactured and used in different major parts (door panel, interior panels, whole body parts, etc.) of cars or other applications, which were previously made from glass fiber composites,^5^ with an aim to reduce the CO_2_ emission significantly throughout the different life cycle stages of composite products. Hence, it is obvious now that many research studies are investigating the possibilities of the use of natural fibers as reinforcement elements for structural/semistructural composite applications.^6,7^ Between different natural fibers, recently composite manufacturers are becoming more interested in jute fibers because of their very low-cost production, abundance, and availability with a good supply chain system, similar mechanical properties like others, such as flax fibers, and most importantly, a good opportunity to optimize the fiber properties further for ultimate composite performance enhancement.

For the polymer matrix in composites, both thermoplastic and thermoset polymers can be used. Thermoplastic polymer matrices (for example, polypropylene, polyethylene, polyester, polylactic acid, polycaprolactone, polyamide, and many others) offer some advantageous properties compared to thermoset polymer matrices. The most important advantage is to have better end-of-life disposal options after their uses since they can be recycled easily and some of them are biodegradable and/or compostable also. In addition, a wide range of thermoplastic polymers is available with a varied range of thermal, mechanical, and thermomechanical properties, which is useful to select and apply a thermoplastic polymer matrix based on the ultimate required properties of composites.

The mechanical properties of the composite materials are the main important issue with employing natural fibers as reinforcements in thermoplastic composites. Generally, traditional textile architectures such as woven and nonwoven (needle-punched) are lacking to satisfy the requirements of high mechanical properties of ultimate composites as they offer very low tensile and flexural properties.^1,8−10^ Two major factors are working actively in reducing the mechanical performance of the composites: the first one is a worse fiber-matrix adhesion and the other one is a low fiber volume fraction of composites. In addition, the fiber placement technique is also a critical factor in designing a composite with enhanced mechanical performance. Ideal options for jute and other natural fiber reinforcements are long fiber derivatives like plain, twill, sateen fabrics, unidirectional sheets, and braided preforms due to their superior formability and exceptional mechanical properties when combined with matrices,^11^ although long fiber-based preforms’ manufacturing process requires several costly and high energy-consuming steps (fiber processing-yarn spinning–sizing–weaving/knitting/unidirectional yarn processing, etc.). As an alternative, short natural fibers including jute, flax, sisal, and hemp have recently attracted increasing interest in thermoplastic composites research for their easy availability and low cost in the preforming process and the possibility of achieving similar mechanical properties to short glass fiber-based composites. The separation of individual fibers from technical fibers, the production of densely packed preforms, and the choice of the fiber length have a significant impact on the load-bearing capacity of short natural fiber composites.^12^

Short natural fiber thermoplastic composites constructed from needle-punched nonwoven preforms have been studied in several studies and published in the literature. These preforms cannot bear sufficient amount of load due to damage of fiber during the needle punching or other steps in the preparation process, as also reported in the literature.^13^ As an alternative route of manufacturing, the natural fiber preform cake formation technique has gained popularity, wherein short-length fibers are placed with homogeneity in the preform. To enhance the composites’ mechanical properties, Bashir et al. investigated how jute fiber length and chemical treatment affected the production of short jute fiber caked-based preforms and their thermoset composites.^8^ Their study indicated that short fiber preforms made from the cake formation technique can increase the fiber content in the composites by more than 15–20%. In this case, fiber individualization contributes to the increase of the fiber packing capacity of the composites, which was also found from the study of Coroller et al.^12^ Hence, there is an option for developing dry short jute fiber preforms with high performance for thermoplastic composites, which is fiber individualization. However, the selection of matrix materials is another crucial factor in composite manufacturing since it affects the stress transmission from the matrix to the fibers as well as the structure of the composite components. Polypropylene (PP), a thermoplastic matrix, is widely used in the automotive and sporting industries.^1^ The use of polypropylene (PP) as a matrix material offers several benefits, including its low processing temperature, which is important for natural fibers, with good thermal stability, mechanical performance, and cost-effectiveness. For these reasons, PP was selected as the matrix material in this study.

Since natural fibers are hydrophilic by nature, their interfacial adhesion with hydrophobic thermoplastics like polypropylene is not good because they include highly polarized hydroxyl groups in their lignocellulosic composition. Weak interfacial adhesion between the nonpolar-hydrophobic matrix and the polar-hydrophilic fibers, as well as poor mixing due to inadequate wetting of the fibers by the matrix, are the key barriers to employ these fibers as reinforcement in such matrices. In order to improve natural fibers’ compatibility and adhesion with the matrix, chemical modification of such fibers is thus required. Some previous studies explained how to reduce the interfacial polarization of reinforcing fibers by doing some chemical treatment to ensure strong interfacial bonding between fibers and matrix.^14−17^ Moreover, it is desirable for the chemical used for modifying natural fibers to preserve their biodegradable properties. One such modification is the alkali treatment, also known as mercerization, which is used for modifying natural fibers like hemp, jute, sisal, and others.^18,19^ Recent studies found that low-concentration alkali treatment with prolonged exposure can not only improve the tensile performance of natural fibers but also significantly enhance the interface quality between the matrix and natural fibers.^20^ In comparison to untreated jute fiber, Roy et al. showed that moderate alkali treatment of jute fiber improved its tensile properties by more than 100%.^21^ Sarker et al. also observed that jute fiber interface quality is enhanced after applying mild alkali treatment due to the parallel printing of fibrils in the elementary fibers after separation of them from the technical fibers.^4^ Therefore, based on prior research, it is anticipated that a moderate alkali treatment at a 0.5% concentration for 24 h can improve both the fiber’s strength and its adherence to the matrix. After alkali treatment, another advantage is to retain the fiber integrity in the compressed preform. Applying matrix-compatible binders such as water-borne epoxy and biocompatible PVA has been successfully used in jute fiber while preparing compressed dry fiber preform both in the long and short form of fiber, respectively.^8,22,23^ A small percentage of binders (PVA sizing) added in the preform retains the structure of dry fiber preform without compromising mechanical properties, while it reinforced with matrix materials.

In this study, highly individualized short jute fiber preforms/PP thermoplastic composites were developed with good mechanical properties, following an easy manufacturing process compared to their conventional preform (long fiber-based unidirectional, woven, etc.)-based composites. According to authors’ best knowledge, this is the first work on developing highly individualized jute short fiber preform composites with the thermoplastic polymer matrix. The alkali treatment followed by a binder application (PVA sizing) was considered in this study as an effective chemical modification of jute fibers for enhancing a good interface between short jute fiber and PP thermoplastic matrix. Therefore, the main objective of this study was to combine the impacts of alkali treatment, binder application, and fiber individualization in the short jute fiber preform manufacturing process to maximize the mechanical properties of short jute fiber/PP thermoplastic composites. Tensile, flexural, and impact testing were conducted to characterize the improvement in mechanical properties. The stiffness and dimensional stability are important for composite materials’ applications, as they determine the ability of a material to retain its shape and resist deformation under load. The intrinsic Young’s modulus of the fibers, which can be determined as the average slope of the stress–strain curve in the strain range of 0–0.3%, is a critical factor in determining the elastic properties of composite materials. The stiffness of the fibers is affected by chemical modification, and measuring the actual stiffness of the fiber is necessary to predict the stiffness of the composite material. In this study, the stiffness of newly formed short jute fiber polypropylene composites was mathematically predicted using the Halpin–Tsai equation and the modified rule of mixing (Cox–Krenchel model),^24,26^ and the tensile modulus was compared to several micromechanical models to determine the best fit for the experimental data.

## 2 Results and Discussion

### 2.1 Physical Properties of Fibers

**Figure 1:**
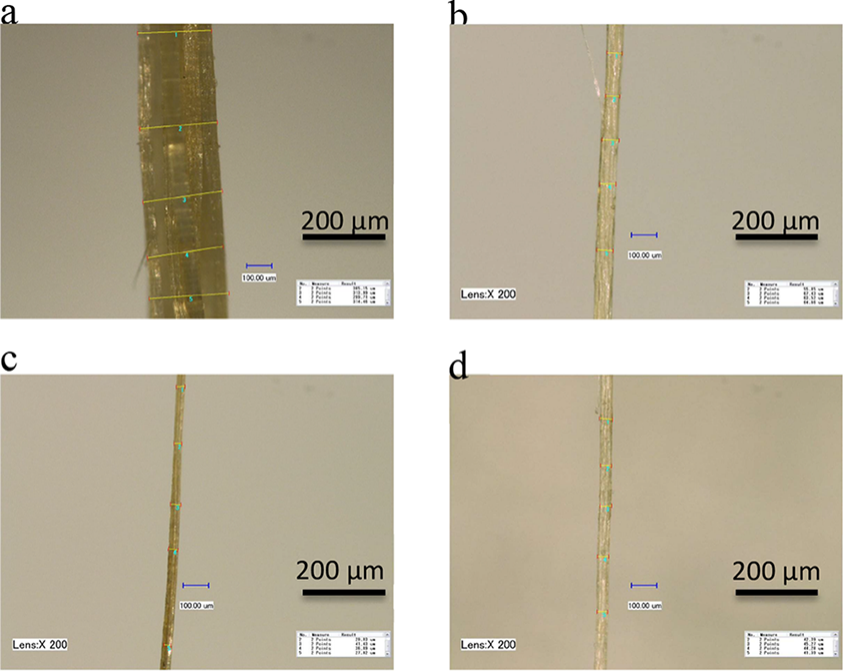
Optical micrographs of jute fibers (200×) used in the study: (a) field retted raw jute fiber; (b) raw jute fiber after mechanical extraction and individualization; (c) jute fiber after being alkalized (AT) and (d) jute fiber after alkali and PVA sizing (AT-sized) application onto it.

**Figure 2:**
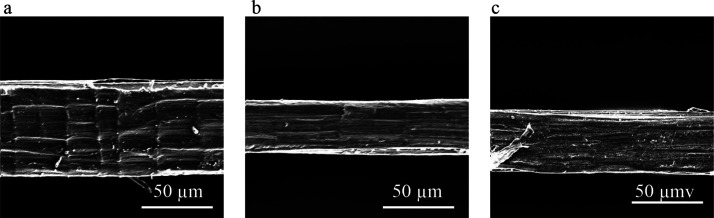
(a) SEM image of field retted jute fiber at 500×; (b) SEM image of alkali-treated fibers after separation from technical fiber at 500× and (c) SEM image of binder-treated single jute fiber at 500×.

Fiber morphology study has been considered one of the important parameters for natural-based reinforcing materials suitable for composite applications. A high-magnification optical microscope with 200× magnifications and a scanning electron microscope (SEM) with 1000× were used to investigate the morphological changes in the fiber. As seen in Figures 1a and 2a, the presence of numerous impurities, such as lignins, wax, pectin, and hemicelluloses, which are situated on the surfaces of the jute fiber, intercell, and interfibrillar area of the fiber, is attributed to the greater diameter of untreated fibers. These major impurities thus may reduce the contact area between fiber and matrices during impregnation. After individualization of jute fibers, a large reduction in the diameter was observed (see Figure 1b). This was because the repeated hand combining of fibers allowed separation of elementary fiber (single fiber) from the technical raw fiber. The pin of the comb helped in removing the binding energy of elementary fibers from the raw fibers originating from the hemicelluloses present in fibers.

When fibers were treated with 0.5% alkali solution, the diameter of the fibers was further reduced slightly and uniformity of fiber along the length of fibers was improved, which might be the result of dissolution of hemicelluloses in jute fiber and removal of lignins from the surface of fibers (see Figures 1c and 2b). As result of this, the treated jute fibers appeared with a rough surface; this can increase the surface area between the fiber and polymer matrices and improve the stress development during tensile loading of fibers. After applying PVA sizing onto alkali-treated jute fibers, no significant changes in fibers were observed, which means sizing treatment has little or no physical effect on jute fibers (see Figure 2c).

### 2.2 Tensile Properties of Composites

**Figure 3:**
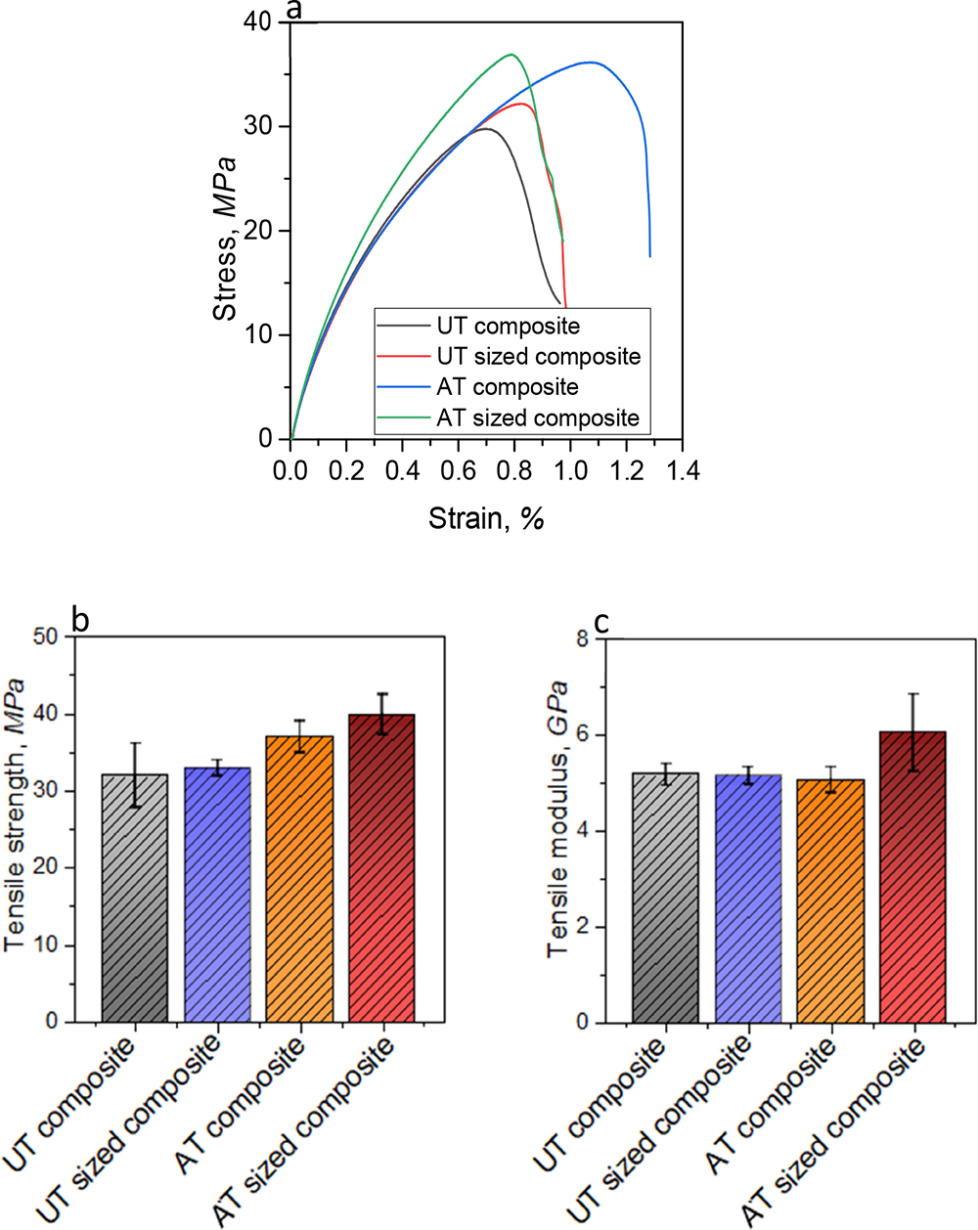
(a) Tensile stress–strain curve for all composites; (b) comparison of tensile strength; and (c) comparison of tensile modulus between treated and untreated composites.

This section discusses the tensile properties of the developed short jute fiber preform-based PP composites. After testing composites, tensile strength and tensile modulus were calculated from the stress–strain curve. Five specimens for each sample types were tested, and data were statistically analyzed. Figure 3b,c shows the tensile properties (strength and modulus) of the tested composites, whereas Figure 3a shows typical stress–strain curves for composites.

Thermoplastic composites developed from short-length jute fibers are not brittle and tend to show viscoelastic behaviors. From Figure 2a, it can be found that composites (UT composite) made from the individualized with a 5 mm fiber length showed a tensile modulus of 5.18 GPa and a tensile strength of 32 MPa only.

As the fiber content in the developed composites was relatively higher and the role of fiber in the matrices had improved due to the interfacial interaction between them, this might have allowed the composites to carry a maximum amount of load than the traditional fabric featured thermoplastic composites. It is exciting and promising that the tensile modulus or stiffness of the developed short fiber composites significantly are higher than the stiffness of other fabric and fiber-architecture-featured composites obtained from the literature and they are reported in Table 4. A possible reason of the better mechanical properties of newly developed composites could be the lower void content in their structures. Newly developed composites used showed very low void contents (below 1%) which ensured improved stress resistance in the composites upon testing. As explained earlier that jute fibers used in the preform are highly individualized, the uniformity of the fiber enhanced, which actually improved the mechanical interlocking between the polypropylene matrix and jute fibers. Adding sizing materials in the raw fiber preform does not change the properties of composites (UT-sized composite) except retaining the structural integrity of the dry fiber in the preform. However, due to the alkali treatment in AT composites, tensile modulus and strength values were found to increase up to 6.05 GPa and 39.9 MPa, respectively, which was around a 25% increment compared to the untreated composites (UT) made from the similar just short fiber length. The findings demonstrate that the alkali treatment on untreated jute fiber for a prolonged period of 24 h with a 0.5% NaOH solution increased the compatibility between the hydrophilic jute fiber and the hydrophobic polypropylene matrix. Mild alkali treatment in this regard ensured better fiber surface by removing the hemicelluloses present in the interfibrillar network of jute fiber also considered for the stress concentration of individual fiber during loading. Lignins present in the intercell of the fiber remain unaffected; as a result, stiffness of the composites showed the maximum value in this study. As shown in Figure 3a, it is also seen that AT composites showed higher modulus and strain values, whereas AT-sized composites exhibited slightly higher tensile strength with a lower strain value. This could be due to the extra treatment with PVA sizing after alkali treatment, which made the preform structure tighter and helped increase the tensile strength, while reducing the breaking extension capability of short fiber composites.

### 2.3 Flexural Properties of Composites

**Figure 4:**
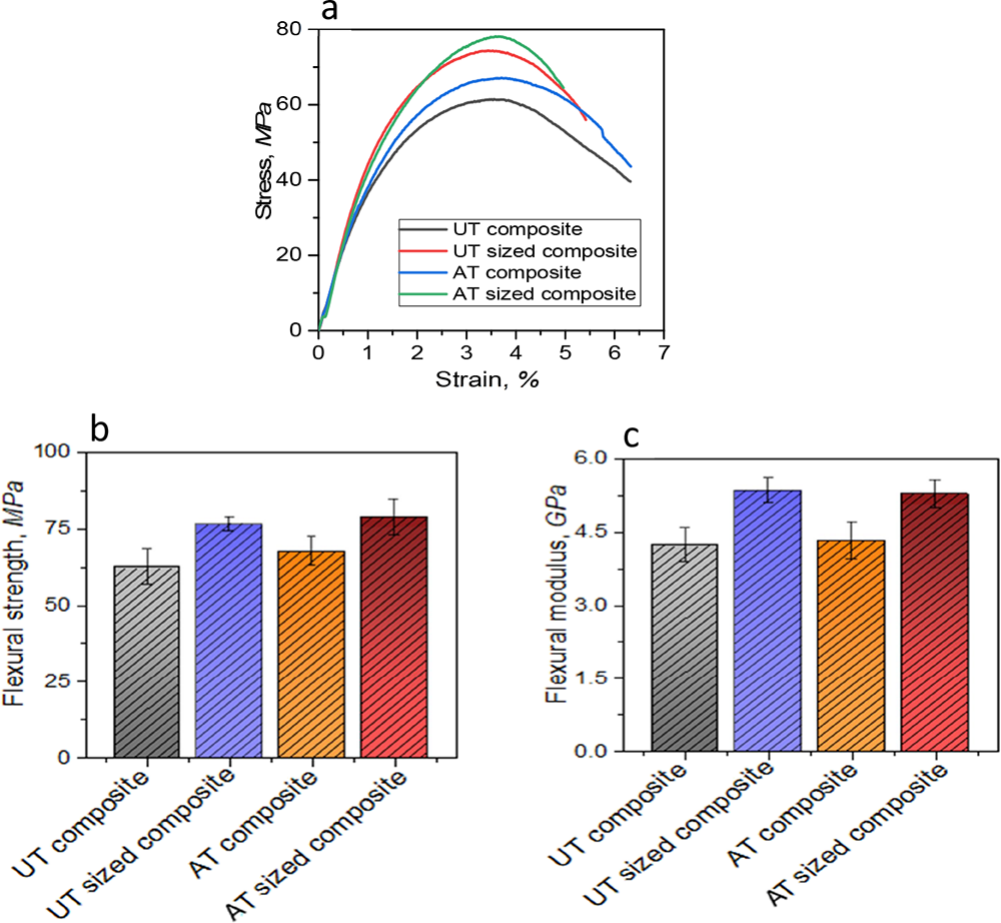
(a) Flexural stress–strain curve for all composites; (b) comparison of flexural strength; and (c) comparison of flexural modulus between treated and untreated composites.

Figure 4a shows typical stress–strain curves for each of the four types of composites, and Figure 4b,c, respectively, compare the properties. All of the composite materials initially failed due to the bottom layers’ tensile yielding, and then, the crack propagated through the layers’ thickness to the top layers of fibers. It is noteworthy that composites were not seen to fail catastrophically. The strong contact between the newly developed fiber and the polypropylene matrix prevented a rapid fracture propagation. For example, composites made from raw fiber (UT composite) displayed a flexural strength of 62.4 MPa and a flexural modulus of 4.2 GPa only. Also, no significant change was found after applying alkali treatment on jute fiber for AT composites (see Figure 4b,c).

**Figure 5:**
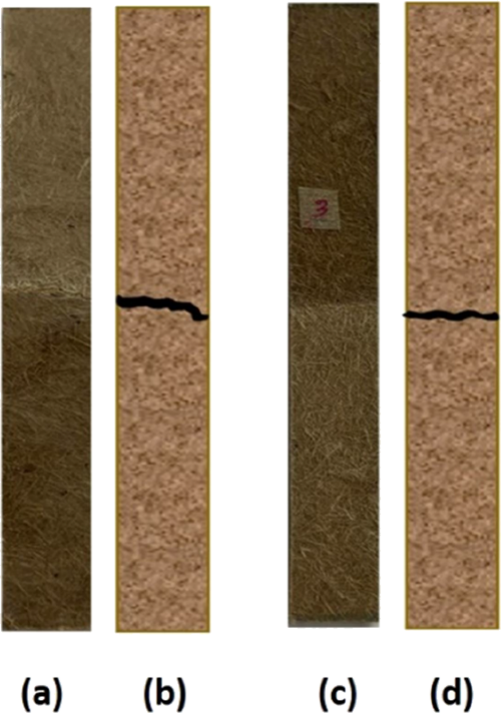
Composite sample failures after the flexural test: (a and b) UT composite real failure and illustrated failure, respectively; (c and d) AT composite real failure and illustrated failure, respectively.

A significant improvement of flexural properties was visible only after applying sizing treatment in both alkali-treated (AT-sized) and untreated jute fibers (UT-sized) preform-based composites. This supports the earlier observation in tensile testing results for PVA-sized tight structure in developed preforms. Flexural strength was achieved up to 78.65 MPa and modulus 5.3 GPa (see Figure 4b,c) for AT-sized composites. This could be due to the synergistic effects of alkali and sizing treatments, leading to the enhancement of the interfacial adhesion between highly individualized fibers and matrix, and this allowed more resin impregnations during the manufacturing process of composites. In addition, sizing helped retain the alignment in individualized jute fibers during processing, which ultimately increased the resistance of composites in bending loading conditions. A similar observation was also reported in our previous published work with the thermoset epoxy matrix. By considering the above flexural behavior of the composites, it can be said that in newly developed jute fiber preforms after chemical treatment and mechanical extraction, it is possible to increase the mechanical load and decrease the possibility of debonding and pull out of fiber from the matrix during the loading conditions. The flexural failure behavior of the composites can be seen in Figure 5a,b along with a schematic representation of failures. Untreated composites (UT) showed an irregular crack propagation (see the black line in Figure 5a,b and in Figure 5c,d); AT composites provided an even crack propagation, indicating better load-bearing capacity under loading.

### 2.4 Fractographic Observations of Composites

**Figure 6:**
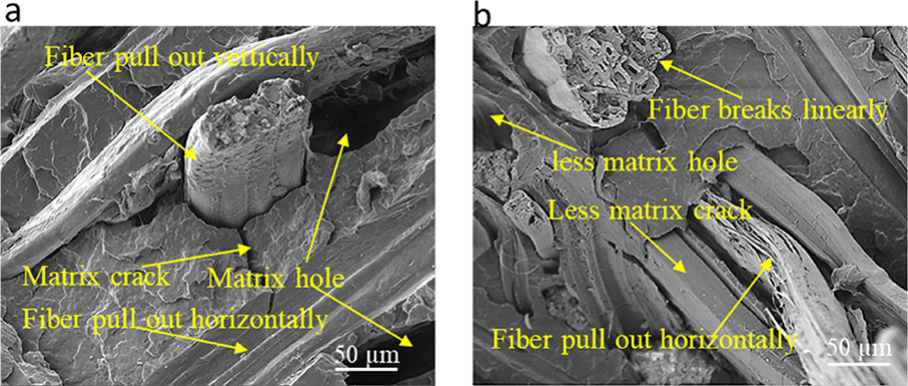
(a) SEM micrograph of the tensile broken sample of composites made from untreated short jute fiber (UT composite) at 1000× magnification and (b) SEM micrograph of the tensile broken sample of composites made from alkali-treated composites (AT composite) at 1000× magnification.

Among four composites, two of the specimens were selected for fractographic observations in order to consolidate the understanding about the interfacial bonding between the fiber and polypropylene matrix. Figure 6 illustrates the SEM micrographs of tensile broken samples of untreated (UT) and alkali-treated (AT) short jute fiber polypropylene composites. Arrow marks in yellow color have been used in the graphs to indicate and identify the status of fiber and matrices inside the composites. For example, Figure 6a shows the fractographic sample of UT composites, where a poor interaction between the fiber and matrix materials was observed. Fiber pull-out with impurities onto the surface of the fiber, matrix hole, and matrix crack at around the fiber indicates that untreated fiber present in the composites is not capable of uniform transferring of stress from the matrix to the fiber to ensure a strong interface in the composites. In contrast, AT composite in Figure 6b shows that fibers were not pulled out vertically, rather fibrils in the fiber were broken linearly during the testing, leading to the presence of a less amount of matrix holes and matrix cracks in the composites. This confirms the removal of polysaccharides present in the fiber after the alkali treatment. Therefore, from the SEM image analysis, a uniform stress development of AT-sized composites was noticed in the tensile testing.

### 2.5 Impact Properties

**Figure 7:**
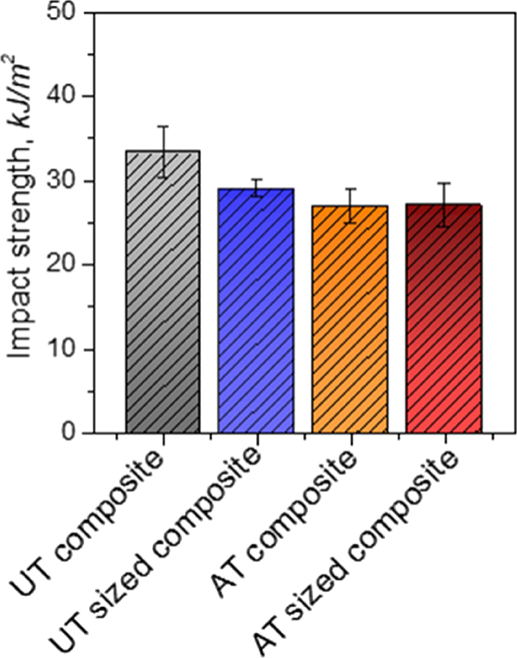
Impact strength of newly developed jute fiber polypropylene composites.

To determine the amount of energy required to fracture the specimens in a sudden impact, Charpy impact tests of composites made from newly developed jute fiber polypropylene were performed. Brittle and ductile nature of the materials can be determined by this test, which ultimately indicates the toughness of the materials. A comparison of different composites made from the newly developed jute fiber is reported in Figure 7.

The Charpy impact strength of the composites made from short jute fiber with modifications behaved differently than the flexural and tensile characteristics. For instance, highest impact strength value was observed for untreated jute fiber polypropylene composites (UT composite) than the treated composites. Average impact strength for untreated composites is found to be 33.33 kJ/m^2^, whereas treated composites (AT-sized composite) made from short jute fiber with sizing materials showed only 26.77 kJ/m^2^. Crack initiation and crack propagation are mainly dependent on the stored energy of the materials, which ultimately depends on the matrix and fiber interactions commonly known as interfacial adhesion.^27,28^ In this case, untreated jute fiber comes with a lot of polysaccharides (waxes, hemicelluloses, lignins, etc.) which may be responsible for consuming more impact energy compared to other composites. Whereas treated composites can initiate and propagate cracks more readily due to cleanliness and uniformity of fibers along the length. Thus, a good interfacial bond by chemical treatment reduces the impact strength of the composites and the serrated failure mode absorbs more energy than the catastrophic failure mode. This statement also agreed with the previous reported natural fiber composite impact strength before and after chemical modifications of the fibers.^1^ However, the reported highest impact strength value is also observed to be maximum for the developed short fiber composites in this work compared to the traditional textile architecture-based jute and other short fiber composites reported in the literature (see Table 4).

### 2.6 Theoretical Analysis of Short Jute Fiber PP Composites

#### 2.6.1 Composite Density and Fiber Volume Fraction

Composites’ fiber volume fractions were calculated based on the rule of mixture formula (weight of fiber, matrix, and composites).^77^ The mass of the fibers (*W*_f_) was divided by the total mass of the composite (*W*_c_) to determine the fiber volume fraction. The volume of fibers, matrix, and voids in the composite, expressed as *V*_f_, *V*_m_, and *V*_p_ respectively, were determined based on these values. The density of the composite material affects its mechanical properties and can be altered by changing the density of its components, the fibers, and matrix. Using eq 2, the density of the composite can be calculated by considering the density of its components
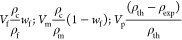
1
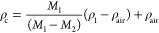
2where “*w*” and “ρ” represent weight and density, respectively, and the subscripts “f”, “m”, and “c” indicate fiber, matrix, and composite, respectively. The density of the fibers (ρ_f_) and the composite (ρ_c_) were measured according to the ASTM-D3800-99 standard using an AJ5OL analytical balance (Mettler Toledo, UK). The density of the composites was calculated by weighing them in air and liquid and using the following formula: ρ_l_ is the density of the liquid, ρair is the density of air, *M*_l_ is the weight of the sample in air, and *M*_2_ is the weight of the sample in the liquid.

Table 1 shows the void content of highly packed short jute fiber composites manufactured, wherein very low void content ranges from ∼2–5% are seen for all composites. The void content of individualized fiber composites (UT) was decreased further when composites were treated specially with alkali treatment and chemically cleaned up. Although fiber volume fraction increased after this judicial impact on jute fiber, the void content significantly reduced.

**Table 1:** Composites’ Density and Void Content

composite sample	fiber volume fraction (*V*_f_)	experimental density (g/cm^3^)	theoretical density (g/cm^3^)	void content (%)
UT composites	40	1.21	1.260	3.95
UT-sized composites	39	1.22	1.271	4.78
AT composite	45	1.23	1.258	2.23
AT-sized composite	45	1.22	1.259	3.10

#### 2.6.2 Stiffness Prediction of Composites

The rule of mixture (ROM) formula is a widely used mechanics formula to predict the stiffness of both natural and synthetic fiber-reinforced composite materials. In this work, the stiffness of short jute fiber composites was predicted using eq 3. In this equation, *E*_c_ is the calculative tensile modulus of composites, *E*_f_, *E*_m_ is the tensile modulus of fiber and matrix, σ_f_ is the strength of the fiber, *V*_f_ and *V*_m_ are the volume fraction of fiber and matrices, respectively. While using this formula, it is believed that all the fibers are placed in the parallel direction and all the stress is developed along the length of the fiber placed in the composites. Interfacial bonding between the fiber and matrix is perfectly fine and the manufactured composites are free from any voids.^29^ Due to the inherent structure of natural fibers, it is always difficult to place all in parallel directions. Moreover, natural fibers always come with a certain percentage of flaws (polysaccharides). As a result, the ROM could not provide accurate predictions for the properties of natural fiber-reinforced composites. Besides the orientation of fibers, there are other important factors also actively responsible for accurately assessing the stiffness of natural fiber composites. These include the length of fibers (critical and original lengths), the actual diameter of the fiber, and the ratio between the length and diameter of the fibers. These variables’ impacts on measuring the stiffness of composites have been reported in previous studies.^30−33^ Considering the orientation of fibers and the efficiency of the length of fibers in the composite, eq 4 can be used. In this study, the critical fiber length was found to range from 3.5–3.84 mm, which indicates an increase in fiber uniformity or a reduction in the fiber diameter (as seen in Table 2). Ideally, the critical fiber length reported in the literature is found to be very low 0.28–0.52 mm.^18^ A few studies reported in the literature reported higher than the usual fiber critical length. This might be related with the process of jute fibers in different mechanical processes involved in the earlier literature studied fibers, which may degrade the quality of fibers and thus reduce the critical fiber length, whereas fibers used in this study obtained from the field without any further mechanical action and thereby critical fiber length is higher than the usual value reported in the literature.
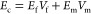
3
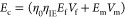
4

**Table 2:** Physical and Mechanical Properties of the Jute Fiber Utilized in This Research

fiber types	tensile modulus of fiber (GPa)	tensile strength of fiber (MPa)	modulus of the matrix (GPa)	tensile strength of the matrix (MPa)	length of fiber (*l*_f_)	diameter of fiber (*D*)	aspect ratio of fiber (*L*_f_/*D*)	critical fiber length (*l*_c_)	length efficiency for stiffness (η_IE_)
untreated jute	30	295	1.3	30	5	48	104	3.50	0.997
untreated sized jute	29	290	1.3	30	5	49	102	3.55	0.997
alkali-treated jute	38	480	1.3	30	5	32	156	3.84	0.997
alkali-treated sized jute	37	475	1.3	30	5	32	156	3.84	0.997

Here, η_0_ = orientation factor and η_IE_ = length efficiency factor
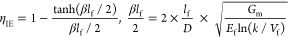
5

Here, *l*_f_ = effective fiber length in the composite, *D* = measured fiber diameter, *G*_m_ = shear modulus of matrix, *E*_f_ = fiber modulus measured, and *K* = constant for fiber packing in the composites.
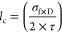
6

Here, τ = is the interfacial shear strength of the composites
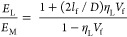
7
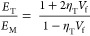
8
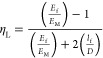
9
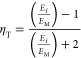
10
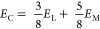
11

The Cox shear lag model is utilized to determine the reduction in the effective length of a composite beam due to transverse shear stress. It is often used to calculate the length efficiency factor for the stiffness of composite beams (η_IE_).^31^ Length of fibers, volume fraction of fibers, tensile modulus of treated and untreated fibers, and tensile modulus of the matrix are reported in Table 2. Besides these variables, fiber arrangements in the composites which is also known as the packing capacity of fibers (constant *K*) has been considered in this study. It is assumed that all the fibers are arranged in the parallel form, and therefore, a constant *K* value is taken as 0.785 which is similar to that of other studies found in the literature.^18,30^ The length efficiency factor calculated in this study was 0.997. Considering the high aspect ratio of the fiber length efficiency factor found in this study also agreed the previous study based on natural fibers.^4,18^ The fiber orientation factor (η_0_) recommended by the study of Krenchel^25^ was considered in this study in order to justify the fiber placement technique effect on composite properties. This value was 0.385 for 2D random fiber orientation reported in the literature by a similar study.^34^Eq 3 has been employed to predict the stiffness of composites considering the length efficiency and orientation factor of fibers in the composites. However, these models are very popular for long fiber-reinforced composites. On the other hand, the Halpin–Tsai model is widely used to calculate the stiffness of short fiber-reinforced composites (see eq 11), where *E*_L_ and *E*_T_ are the predicted tensile modulus of short fibers in the longitudinal and transverse direction. The modulus of fibers in different directions can be found by using eqs 9 and 10.

Theoretical tensile modulus prediction based on ROM, Cox–Krenchel (MROM), and Halpin–Tsai model and experimental results are compared in Table 3. It is observed that the tensile modulus of the composites increases with the change of chemical modification, and it follows a same trend for all models. However, simple of ROM formula is not best suitable for this kind of novel short fiber composites as it does not follow the conditions explained in the earlier section. However, theoretical results obtained from MROM and the Halpin–Tsai model is closed to the experimental results (see Table 3). It is worth noting that after applying chemical treatment and fiber individualization, the tensile modulus of composites best fitted theoretical results but better adjusted with the Halpin–Tsai model.

**Table 3:** Comparison of Jute/PP Composite Stiffness Experimental Results with Micromechanical Model Data

composite type	fiber volume fraction (*V*_f_)	experimental result (GPa)	rule of mixture equation (GPa)	modified role of mixture equation (GPa)	Halpin–Tsai equation (GPa)
untreated jute composites (UT)	40	5.18	12.50	5.30	7.20
untreated sized jute composites (UT-sized)	39	5.15	12.40	5.22	7.25
alkali-treated jute composite (AT)	45	5.07	17.80	7.20	7.17
alkali-treated sized jute composite (AT-sized)	45	6.05	17.8	6.99	7.09

This comparison of study clearly indicates that composite property enhancement is not only related with the performance of reinforcing fibers but also with a lot of factors related with matrix used in the composites. The composite manufacturing process, chemical treatment, mechanical extraction of fibers significantly contributed to the quality of composites by enhancing the volume content. In the case of this study, tensile modulus of AT and AT-sized composites showed higher fiber content and best fitted with the theoretical results. As shown in Table 4, a comparison is presented between the mechanical performances of newly developed short jute fiber/PP composites in this work and other traditional structured jute fiber composites found in the literature. A clear improvement is seen for the newly developed short jute fiber-based preform thermoplastic composites. This is considered potentially advantageous to explore and expand the application of this new short fiber preform-based thermoplastic composites in various mechanically demanding applications.

**Table 4:** Comparison of Mechanical Properties of Short Jute Fiber Polypropylene Composites with the Literature^a^

composite type	fiber type	tensile strength (MPa)	tensile modulus (GPa)	flexural strength (MPa)	flexural modulus (GPa)	impact strength (kJ/m^2^)
jute/epoxy^35^	short 30 W	32.90	0.054	88.31	7.620	3.8097
jute/epoxy^36^	short 50 W	16.69	0.66			13.44
jute epoxy^8^	Short 50 V	34	4.4	110	5.5	
jute/epoxy^8^	mat 50 V	28	1.8	35	2	
banana/epoxy^37^	woven 60 W			28.18	2.68	
jute/polyester^28^	woven 50 W	49.9	2.20	55	1.6	10
Jute/PP^1^	mat 40 V	25	3.6	26	2.7	14
kenaf/PP^1^	mat 40 V	29	6.8	28	2.3	15
kenaf/PP^38^	mat 35	28	1.4	32		
jute/PP^39^	woven	20.1	1.25	52	4.05	19
glass/PP^1^	mat 22 V	88.6	6.2	60	4.38	54.16
jute/PP	short 45 V	39.9	6.03	78.5	5.3	32

a *W* stands for weight fraction of jute fiber and *V* stands for volume fraction of fibers used in composites.

## 3 Conclusions

In this study, the mechanical properties of newly developed short jute fiber preform polypropylene thermoplastic composites were developed, characterized, and the tensile modulus was theoretically predicted using a modified rule of mixing formula.

- The new manufacturing process for short jute fiber composites presented in this work was able to individualize fibers from technical fibers significantly. In addition to this, the applied chemical treatments increased the fiber volume fraction and adhesion between the fiber-matrix interface, which eventually improved the mechanical properties of short jute fiber PP composites compared to any traditional architecture (woven, nonwoven, unidirectional) jute composites found in the literature.
- Alkali treatment alone increased both tensile modulus and strain values, whereas a combination of alkali and PVA sizing treatments provided a tighter structure in composites which was helpful for increasing both tensile and flexural strength, although the failure strain was found to decrease for the PVA sizing treatment.
- The stiffness of the fibers underwent significant changes after physical and chemical modifications, which played an important role in deciding the fiber’s contribution to the composite’s tensile modulus.
- The simple rule of mixture was not able to accurately predict the stiffness of the composites as it only considered fibers arranged in a parallel direction. The modified ROM considered the orientation factor of the fibers, leading to a better match between the experimental stiffness of the composites and the modeled data.

It is expected that in many semistructural applications, this newly created short fiber-based thermoplastic jute fiber preform composites can be an effective replacement of glass fiber thermoset or thermoplastic composites and hence maintain environmental benefits because of their biodegradability/recyclability option.

## 4 Materials and Methods

### 4.1 Materials

**Figure 8:**
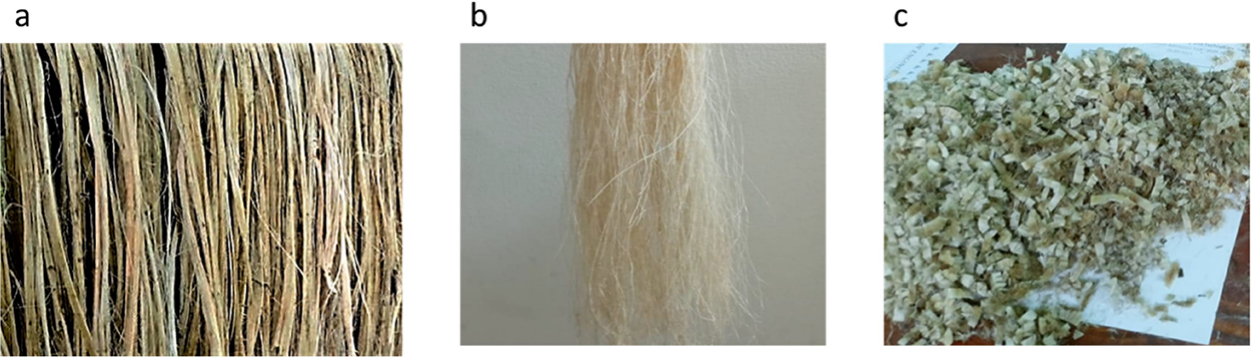
(a) Untreated jute fiber collected from field; (b) individualized jute fiber and (c) cut jute fibers in short-length (5 mm) used in this study.

Field retted jute fiber (coljute fiber, *Corchorus olitorious*), commercial grade-Bangla Tossa Special (BT-S), was purchased from farmers in Dhaka, Bangladesh. The sizing material (polyvinyl alcohol) was supplied by Aristek High Polymer, West Java, Indonesia. Polypropylene (PP) polymer granules (trade name: Cosmoplene) and sodium hydroxide (NaOH) pellets used in this study were purchased from Merck (Germany). Figure 8a displays a digital image of field retted jute fibers. Physical and mechanical properties of the materials used in this study are reported in Table 5.

**Table 5:** Materials Properties Used in This Study^a^

s. no.	materials	viscosity (mPa s)	gel time at 25 °C (min)	cured density (g/cm^3^)	tensile modulus (GPa)	tensile strength (MPa)	tensile strain (%)
01	jute fiber			1.48	28	298	1.12
02	epoxy	800	90	1.15	3.15	70	6
03	PVA binder	1–1.6					

a Provided from the supplier information.

### 4.2 Methodology

#### 4.2.1 Fiber Individualization and Short Fiber Preparation

In this study, elementary fibers were separated from technical fibers by the manual hackling process on a combining device, which is used in the preparatory section of a jute spinning process. The separated individual fiber image can be seen in Figure 8b. The fibers were then cut into short lengths (5 mm) to be processed into a preform (see Figure 8c). The complete process of fiber preparation by mechanical extraction can be found in the authors’ previously published work,^8^ and it was found that 5 mm length fibers provide better mechanical properties in ultimate composites.

#### 4.2.2 Chemical Treatments of Short Jute Fibers

**Figure 9:**
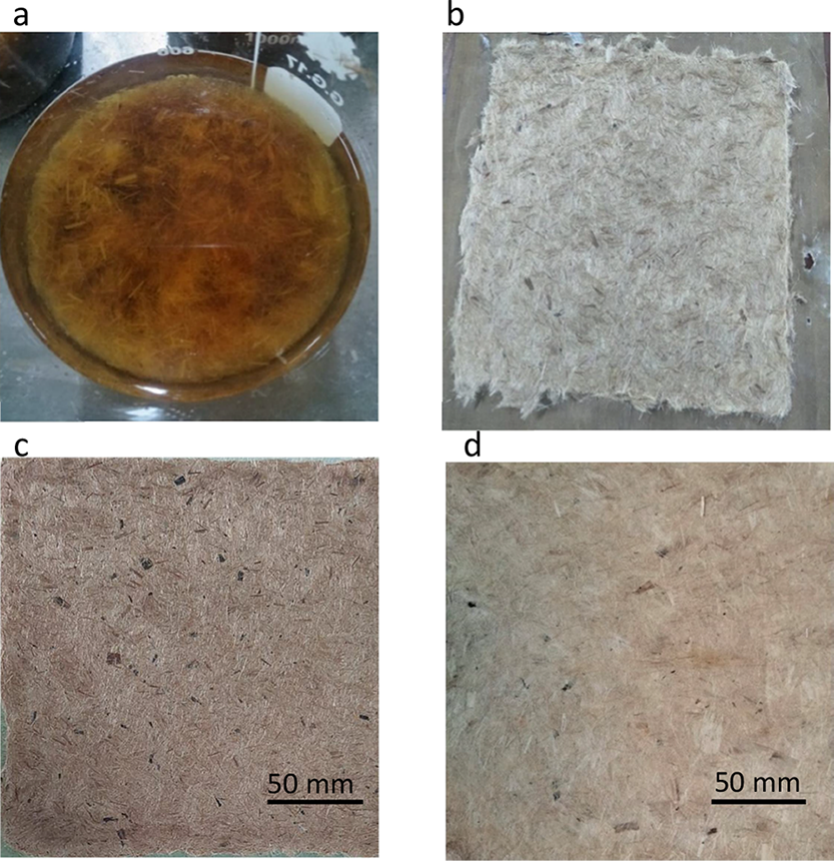
Chemical modification of short jute fiber and their transformation into preform and composite (200 mm × 200 mm) with digital images: (a) alkali treatment on jute fibers; (b) short jute fiber preform after applying sizing materials without drying; (c) highly packed short jute fiber preform after drying; and (d) newly developed short jute fiber polypropylene composites.

In order to remove the polysaccharides, the jute fibers were treated with a 0.5% NaOH solution having a 1:30 liquor ratio at room temperature for 24 h.^8^ Alkali-treated fibers were washed carefully and dried in room temperature for one day. Both alkali untreated (UT) and AT fibers were immersed for 30 min at room temperature in a 1% (W/V) PVA sizing (binder) solution. The UT and AT-sized fibers were collected in a metal box where the bottom part of the box was made of metal wired mesh to rinse sizing water easily, followed by a normal drying process in the room temperature. The metal box helped the fibers to get a shape of sheet of fibers, as shown in Figure 9b,c. Further processing of the treated fibers is explained in Section 4.2.4. PVA-sized jute fibers were named as UT-sized and AT-sized jute fibers. Figure 9a–c displays a digital image of jute fibers after being treated.

#### 4.2.3 Tensile Properties of Jute Elementary Fibers

To use the effective experimental value of the tensile testing data in estimating the stiffness of the composites, the tensile properties of the treated (AT and AT-sized) and untreated (UT and UT-sized) jute fibers were measured. A total of 30 single fibers of each type of fibers were tested for getting the average value of data. Paper card frames were used to place the fiber in a 20 mm cut spaced window where singles fibers were mounted carefully and placed by adding the superglue drop collected from the local market. Samples were kept 24 h for ensuring curing before conducting the test. The test process details are described in our earlier publication.^40^ Results were used to calculate different properties of composites which are provided in Table 2.

#### 4.2.4 Dry Fiber Preform Development

**Figure 10:**
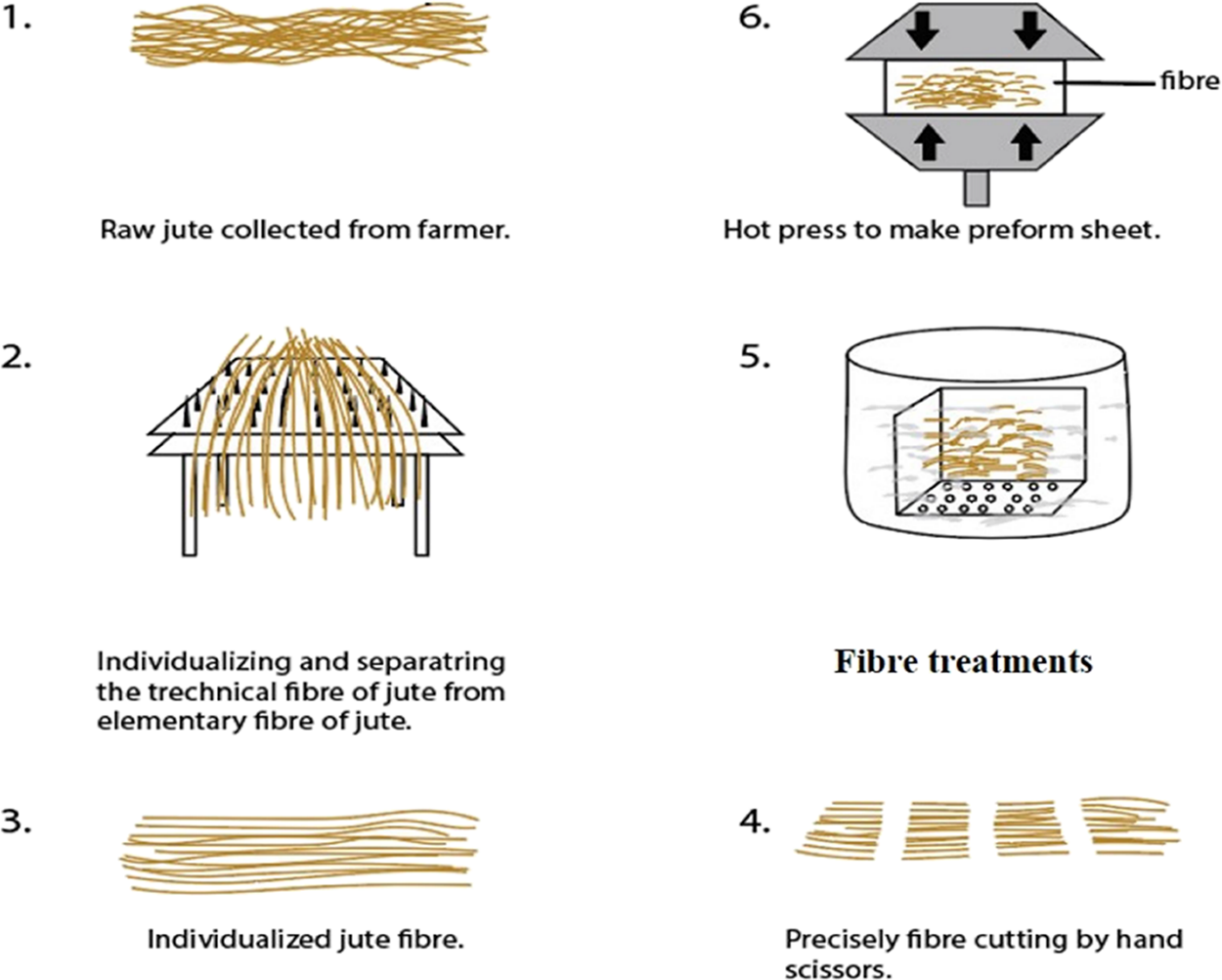
Schematic diagram on the development of preform from short jute fiber: (1) collection of jute fiber; (2) separation of elementary fiber; (3) uniform jute fiber; (4) 5 mm chopped jute fiber; (5) boiling of fiber to manufacture fiber cake; and (6) compaction technique to manufacture preform.

Both dried treated (AT, AT-sized, UT-sized) and untreated (UT) short jute fibers were collected from metal box (see Section 4.2.2) and placed between two platens of the compression molding machine. A pressure of 25 MPa was applied on fibers to compress and transform them into dry short fiber preforms. A schematic diagram of developing preforms can be seen in Figure 10. Original digital images newly developed preform can be seen in Figure 9c.

#### 4.2.5 Polypropylene Sheet Preparation

Polypropylene (PP) sheets were developed by using the compression molding machine. A rectangular metal mold of 200 × 200 mm was fabricated with Teflon sheet first and PP beads were heated at 180 °C for 3 min under 25 MPa e into the mold. As a result, PP sheets were developed and ready to use as the matrix in short jute fiber/thermoplastic composites. The thickness of the PP sheet was measured as 0.5 mm (±0.045).

#### 4.2.6 Fabrication of Composites

**Figure 11:**
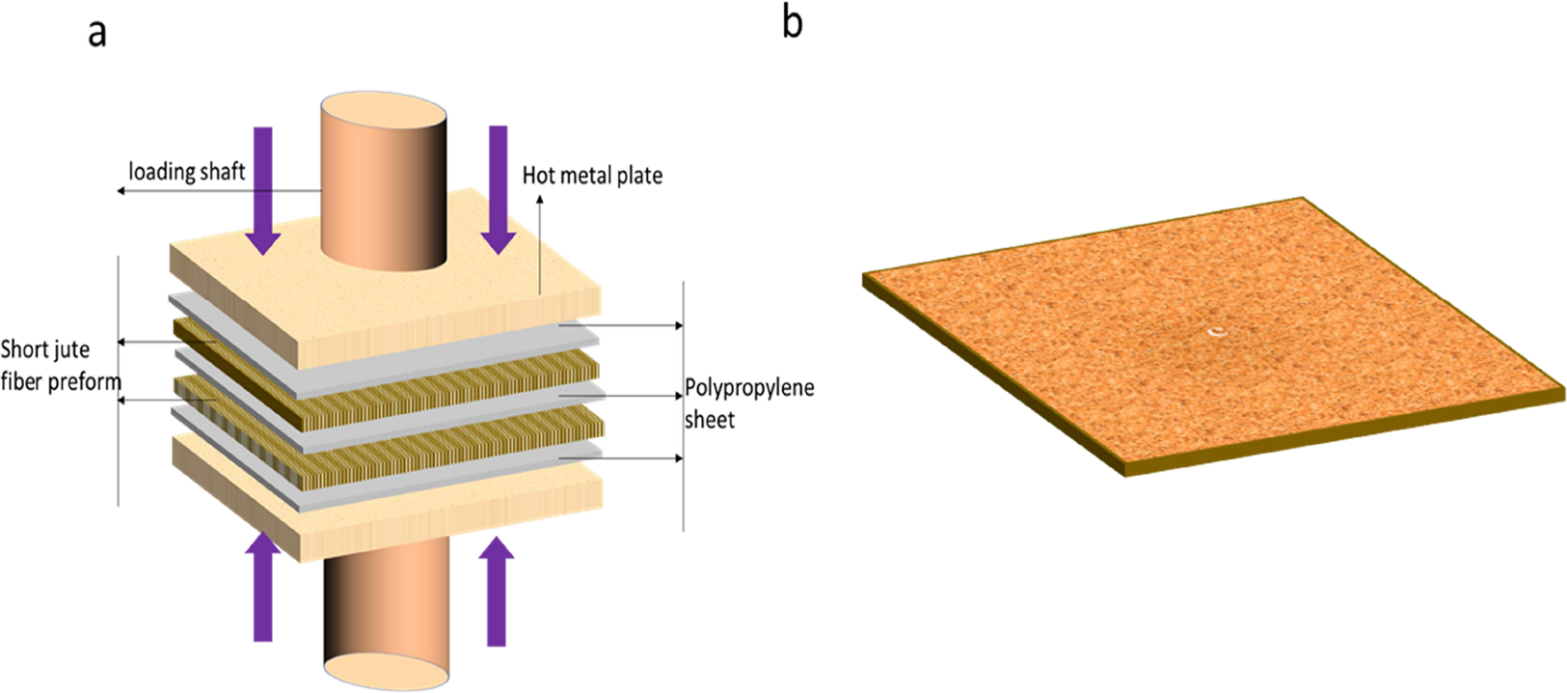
(a) Manufacturing of composite using the hot press machine and (b) produced jute fiber composites after compaction of polypropylene and jute fibers.

Highly packed short jute fiber preforms were stacked with PP sheets to manufacture composites using a hot compression molding machine. A 200 mm × 200 mm sized metal frame with three mm thickness was used as mold for making composites. In the mold, our PP sheets and two dry jute fiber preform were stacked together to manufacture the composites. Composite fabrication was completed at 180 °C and 25 MPa for 15 min. At the end of the process, temperature was reduced to room temperature at 3 °C/min without removing the pressure (see Figure 11a). Composites were named as their preform processing technique employed in the previous section. Hence, the name of the composites is (i) UT composites made from untreated jute fiber, (ii) UT-sized composites made from untreated jute fiber after the application of sizing materials on the fiber, (iii) AT composite made from alkali-treated jute fiber, and (iv) AT-sized composites made from alkali-treated jute fiber after the application of sizing materials on the fiber. Manufactured composites image can be found in Figures 9d and 11b. The thickness of the composites was measured as 3 mm (±15) based on the mold thickness used in this study.

#### 4.2.7 Mechanical Testing of Composites

**Figure 12:**
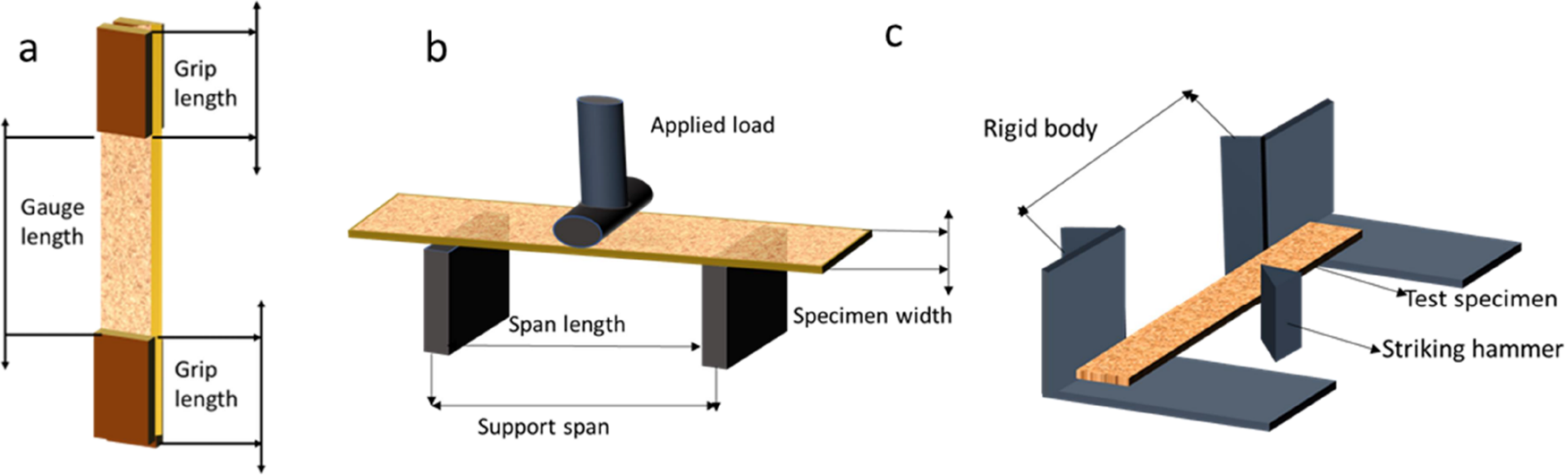
(a) Tensile test specimen; (b) flexural test set up; and (c) impact test set up used in this study.

Tensile, flexural, and Charpy impact tests with unnotched samples were considered in this study to evaluate the mechanical performance of the composites. Figure 12a–c shows a schematic diagram of the tensile, flexural, and impact tests.

##### 4.2.7.1 Tensile Testing

Tensile tests were performed following the ASTM D 638-03 standard using an AG-X plus Universal Testing machine from Japan. The machine was equipped with a 20 kN load cell. Specimens for the tests were prepared with dimensions of 165 mm length, 20 mm width, and 3 mm thickness. The cross-head speed of the machine remained constant at 1 mm/min throughout the testing process.

##### 4.2.7.2 Flexural Testing

Using the same Universal Testing machine (UTM), a three-point bending flexural test was performed. The test was conducted in accordance with the ASTM D 790 testing standard. The specimen dimensions were 125 mm length, 20 mm width, and 3 mm thickness, with a test span of 50 mm. The cross-head speed during the test was set at 1.4 mm/min.

##### 4.2.7.3 Impact Testing

Impact tests were conducted on unnotched composite samples following the ASTM-D256 standard. The samples were vertically positioned, and a 2.634 kg pendulum was released freely to strike the sample at an angle of 150°. An indicator was used to determine the precise angle at which the pendulum contacted the sample. The impact energy was then calculated using a chart provided with the impact tester.
